# Granulomatous cholangitis mimicking hilar cholangiocarcinoma: a case report

**DOI:** 10.1186/s12876-020-01519-1

**Published:** 2020-11-04

**Authors:** Shigeru Fujisaki, Motoi Takashina, Ken-ichi Sakurai, Ryouichi Tomita, Tadatoshi Takayama

**Affiliations:** 1 Department of Surgery, Fujisaki Hospital, 1-25-11, Minamisuna, Kotoh-ku, Tokyo, 136-0076 Japan; 2 Division of Digestive Surgery, Nihon University School of Medicine, 30-1, Oyaguchikamimachi Itabashi-ku, Tokyo, 173-8610 Japan; 3 Department of Surgery, Nippon Dental University School of Life Dentistry, 2-3-16 Fujimi, Chiyoda-ku, Tokyo, 102-8158 Japan

**Keywords:** Granulomatous cholangitis, Hilar cholangiocarcinoma, Fungal infection

## Abstract

**Background:**

Hilar biliary stricture caused by isolated fungal infections in immunocompetent patients are considered to be extremely rare and difficult to the diagnose from the outset.

**Case presentation:**

We report a unique case of granulomatous cholangitis based on isolated biliary fungal infection manifesting as obstructive jaundice and mimicking hilar cholangiocarcinoma in an immunocompetent woman. A 67-year-old Japanese woman was referred to our hospital for obstructive jaundice. She had been followed up for hypochondroplasia by the referring physician. Her total bilirubin level was 5.4 mg/dL. Viral hepatitis screening was found to be negative, and serum IgG4 was within normal limits; however, her CA19-9 level was high. Abdominal computed tomography revealed dilatation of the intrahepatic bile ducts. Abdominal echogram detected a solid mass in the hilar bile duct. Her magnetic resonance cholangiopancreatography has also revealed an abrupt stenosis of the primary biliary confluence with upstream dilatation of the intrahepatic bile ducts. Endoscopic nasobiliary drainage was then performed to improve the obstructive jaundice. Although biliary cytology did not reveal malignant findings, the bile duct in the hilum showed severe stenosis, and hilar cholangiocarcinoma could not be completely excluded. The patient had a developmental disorder based on chondrodystrophy. To avoid excessive surgical stress, such as hepatic lobectomy, we performed resection of the extrahepatic bile duct and Roux-en-Y hepaticojejunostomy reconstruction. Intraoperative frozen sections of the resection margins were determined to be negative for tumor. The resected specimen showed multiple strictures inside the common bile duct, numerous calculi in the lumen, and little free space. The final pathological diagnosis was granulomatous cholangitis due to fungal infection. The patient’s postoperative course was deemed uneventful. She was discharged from our hospital 23 days after surgery without antifungal treatment.

**Conclusions:**

For a unique case of granulomatous cholangitis based on isolated biliary fungal infection mimicking hilar cholangiocarcinoma, we were able to avoid excessive invasion and performed appropriate surgical management.

## Background

Biliary fungal infection has rarely been observed, even in susceptible patients with systemic candidiasis [1]. Moreover, there is little chance of systemic fungal infection in immunocompetent patients, and biliary fungal infection is unlikely.

We report a case in which we performed an operation for biliary stricture in the hilum region because hilar cholangiocarcinoma could not be ruled out. This was an extremely rare case of granulomatous cholangitis based on isolated biliary fungal infection as the final pathological diagnosis in an immunocompetent woman.

## Case presentation

A 67-year-old Japanese woman with chondrodystrophy was referred to our hospital for obstructive jaundice. Her urine color had become darker, and her appetite had decreased during the past 2 weeks.

### Family history

The patient had no family history of hepatobiliary disease.

### Past history

The patient had been followed up with chondrodystrophy for a long time at the previous doctor.

She had no past history of notable medication, no overseas travel history, no history of alcohol/drugs consumption, no exposure to thorotrast and no possible exposure to liver flukes such as Opisthorchis viverini and Clonorchis sinensis.

### Physical examination

The patient had a developmental disorder of chondrodystrophy; her height was 127 cm, and her weight was 37 kg. As a result of her disease, she was found to have weak muscle strength in her lower legs. Palpebral conjunctiva was not anemic, and bulbar conjunctiva was icteric. The abdomen was soft and flat. No physical symptoms associated with immunodeficiency were noted.

Vital signs included a pulse of 86/min, regular blood pressure of 129/59 mm Hg, respirations of 12/min, and temperature of 37.2 °C.

At the time of referral, laboratory analysis showed a white blood count of 14,300 × 10^3^/μL with 35.0% neutrophils and C-reactive protein of 2.78 mg/dL (normal range < 0.50 IU/L). Liver function test showed a total bilirubin of 5.4 mg/dL (normal range 0.2–1.0 mg/dL), direct bilirubin 4.3 mg/dL (normal range 0.0–0.4 mg/dL), aspartate 181 IU/L (normal range 9–35 IU/L), and alanine aminotransferase 265 IU/L (normal range 5–30 IU/L), lactate dehydrogenase 528 IU/L (normal range 106–211 IU/L), gamma- glutamyltransferase 538 IU/L (normal range 10–55 IU/L), and alkaline phosphatase 1232 IU/L (normal range 104–338 IU/L).

Viral hepatitis screening was negative, with an antinuclear antibody titer of 320 (normal range < 40) and negative antimitochondrial antibody. Serum IgG level was normal at 1520 mg/dL (normal range 870–1700 mg/dL) and serum IgG4 level was also normal at 35.7 mg/dL (normal range 4.8–105 mg/dL), whereas the serum CA19-9 level was elevated at 742.2 U/mL (normal range < 37.0 U/mL).

**Fig. 1:**
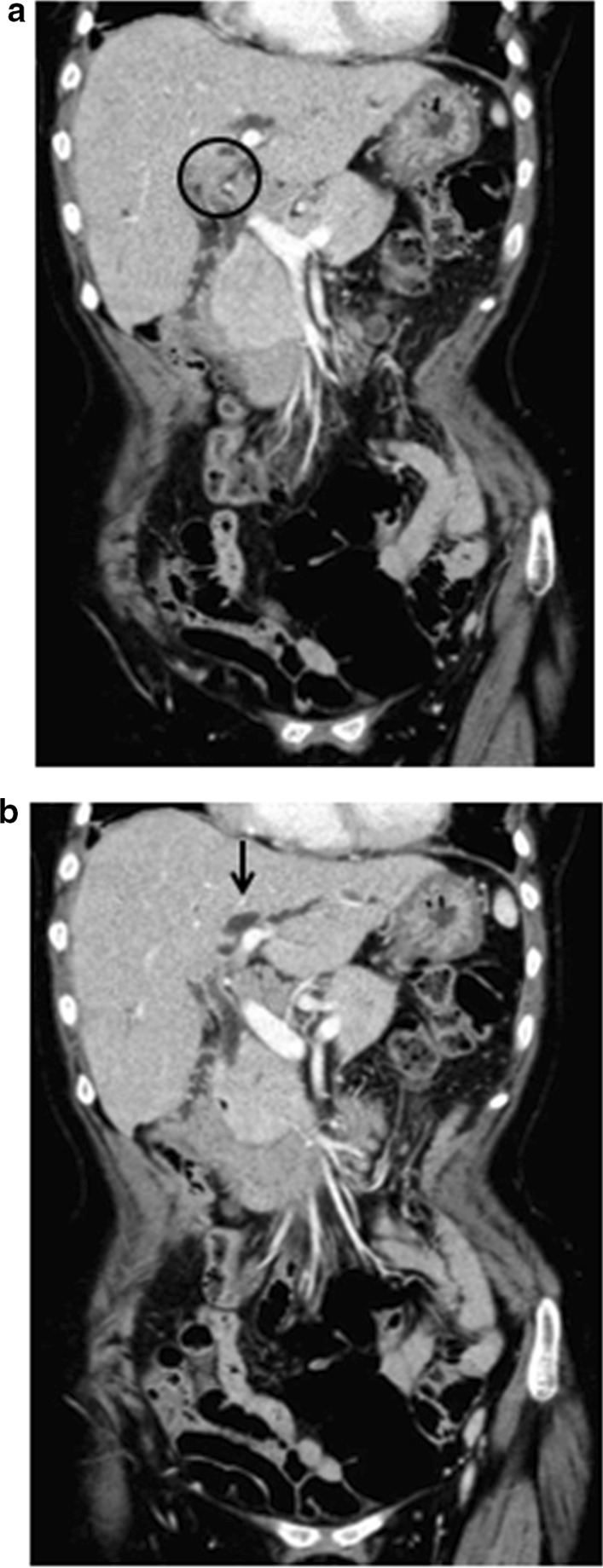
Abdominal computed tomography. **a** Solid mass in the hilar bile duct (circle). **b** Slight dilatation of intrahepatic bile ducts in the left lobe (arrow)

**Fig. 2:**
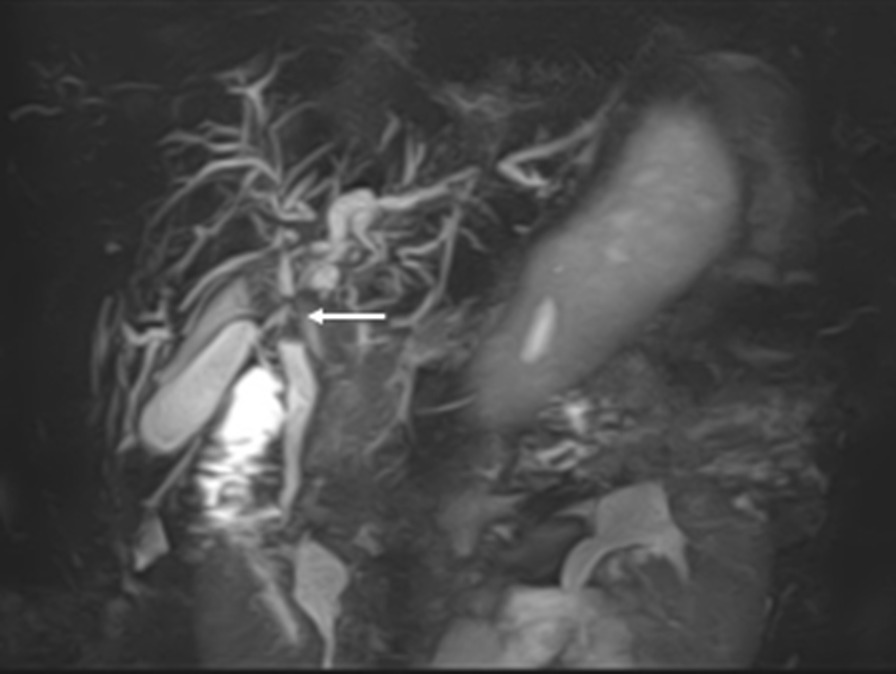
Magnetic resonance cholangiopancreatography. An abrupt stenosis of the primary biliary confluence (arrow)

Abdominal echogram has detected a solid mass measuring 19 mm in the hilar bile duct. Computed tomography of the abdomen revealed a solid mass in the hilar bile duct (Fig. 1a, circle), and a slight dilatation of the intrahepatic bile ducts in the left lobe (Fig. 1b, arrow) was also observed. Abrupt stenosis of the primary biliary confluence was observed on magnetic resonance cholangiopancreatography (Fig. 2, arrow).

**Fig. 3:**
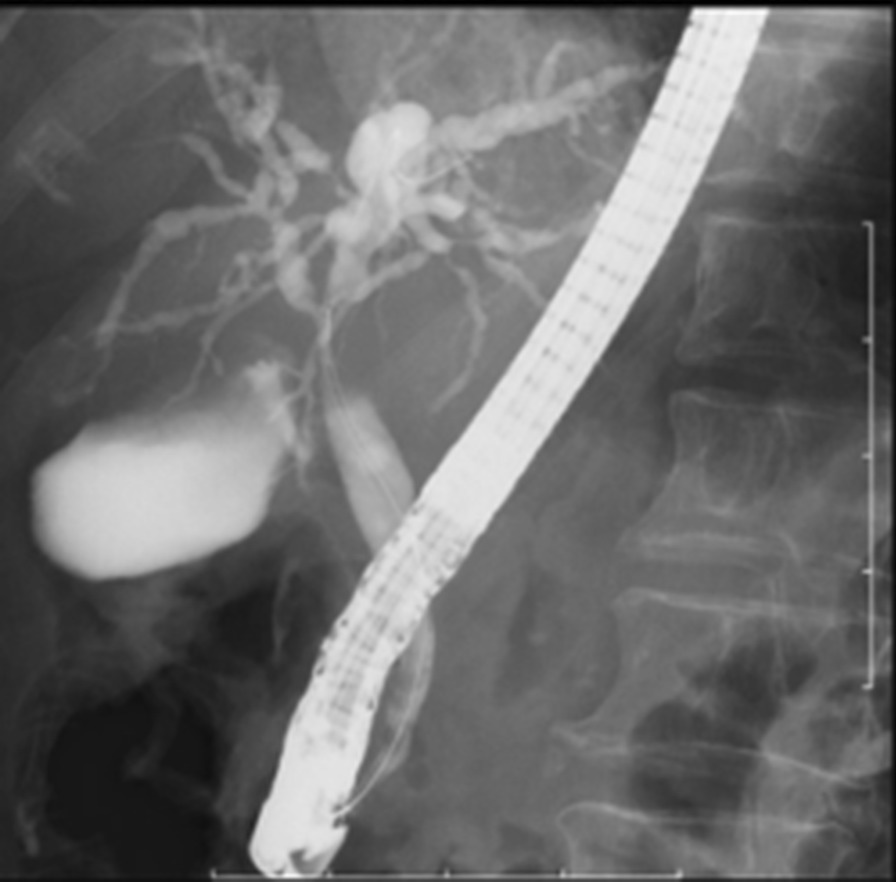
Endoscopic nasobiliary drainage (ENBD). To improve biliary obstruction, an ENBD tube was placed in the left hepatic duct. The patient inappropriately removed the ENBD tube. Thus, the ENBD tube was replaced in the right hepatic duct

In order to improve biliary obstruction, an endoscopic nasobiliary drainage (ENBD) tube was placed in the left biliary duct on the sixth day of hospitalization (Fig. 3). On the 10th day of hospitalization, the patient inappropriately removed the ENBD tube, which was replaced on the 12th day of hospitalization. As a result of the biliary drainage, liver dysfunction was normalized and the inflammatory response also was normalized. No abnormalities were found in vital signs after the drainage. Biliary obstruction was considered to be the main cause of liver dysfunction. The ANA value was high, but there was no abnormality in the IgG level, and there were few clinical findings suggestive of autoimmune hepatitis. Since serum IgG4 was also normal, the possibility of IgG4-related sclerosing cholangitis was unthinkable. Moreover, systemic fungal infection was ruled out by blood culture. Urine culture was also negative.

Biliary cytology failed to detect any malignant findings. Biopsy using endoscopic ultrasound was not desired by the patient due to the risk of complications and cancer implantation. However, abdominal ultrasonography and abdominal computed tomography examination confirmed a solid mass in the hilar bile duct, and cholangiography showed severe stenosis of the bile duct. In addition, serum CA19-9 was found to be elevated at 742.2 U/mL. Thus, the bismuth type II hilar cholangiocarcinoma could not be excluded. Diagnostic imaging showed no findings suggestive of metastasis. Based on the results of the preoperative workup, we considered surgical exploration for a biliary occupying lesion suspicious for cholangiocarcinoma.

### Operation

An exploratory laparotomy was performed 2 weeks after biliary drainage. Thorough peritoneal inspection revealed no metastatic disease. The hilar hepatic duct was determined to be enlarged and clogged in the lumen. After confirming the location of the mass in the biliary tract using intraoperative ultrasonography, we resected the extrahepatic bile duct and prepared the intraoperative frozen section diagnosis from the resected specimen. Using blunt dissection, the gallbladder, cystic duct, extrahepatic bile ducts, hepatoduodenal lymph nodes, and soft tissues were dissected from the portal vein and hepatic artery, which were completely skeletonized. The isolated distal bile duct was then transected on the side of the pancreas away from the bile duct mass. While pulling the distal stump of the bile duct in a ventral direction, the hilar structures (i.e., the biliary confluence and the bases of the left and right hepatic ducts) were exposed. The proximal ducts were then transected 10 mm or more away from the biliary confluence at both right and left hepatic ducts.

The extrahepatic bile ducts including the biliary confluence were then removed en bloc. The thickness of the ductal wall was observed macroscopically throughout the resected extrahepatic biliary tract. A circumferential mass lesion (0.6 cm × 1.0 cm, white, elastic, and hard) was determined in the bile duct 8 mm distal from the biliary confluence. About 10 black bile duct stones, which are 5–6 mm in size, were detected in the lumen of the resected bile duct. The macroscopic appearance of the mass was indicative for a benign lesion, although we could not completely rule out its malignancy.

**Fig. 4:**
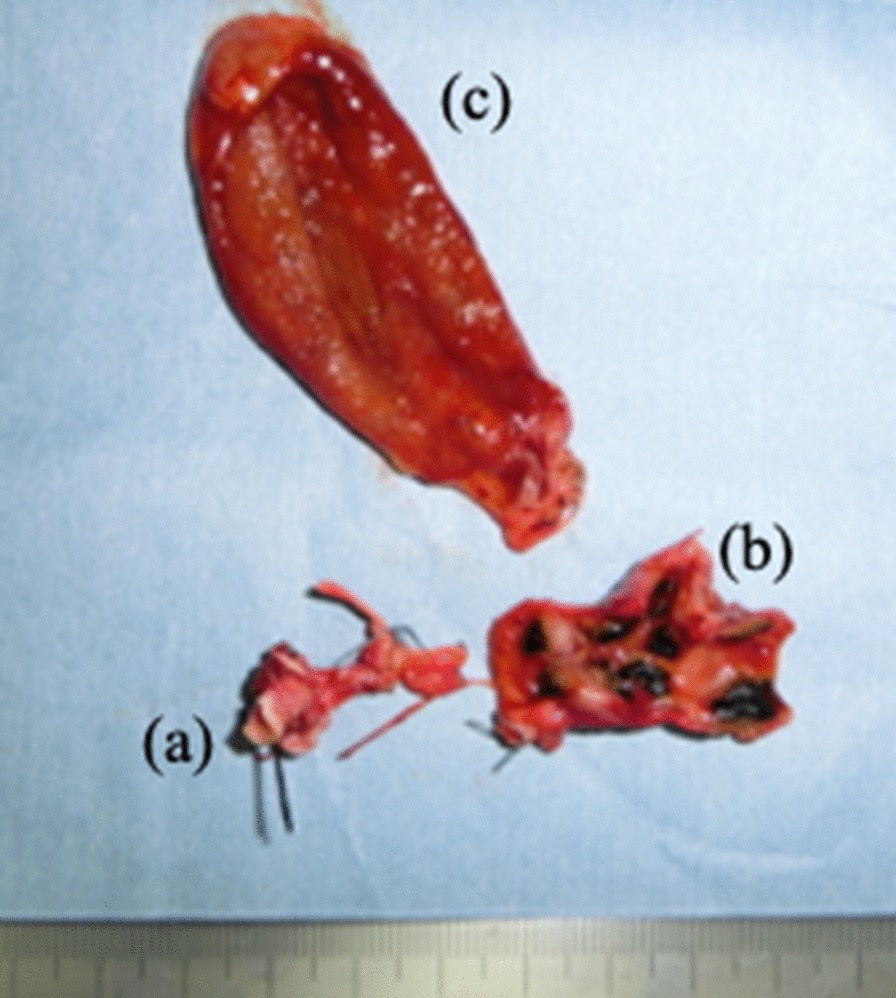
Surgical specimens. **a** Excised stump of the pancreatic side. **b** Extrahepatic bile ducts with multiple stenosis of the lumen, where stones occurred. **c** Gall bladder

From the intraoperative frozen sections of the resection margins, atypical cells were found in the stump on the side of the pancreas, and malignancy could not be ruled out. Thus, we reported the stump as positive. We performed additional excision of the bile duct to just above the pancreatic head. The resected specimens have showed multiple strictures inside the common bile duct, numerous calculi in the lumen, and little free space (Fig. 4). We performed a Roux-en-Y cholangiojejunostomy.

The patient’s postoperative course was deemed uneventful. No other fungal infection focus was observed. The patient was discharged from our hospital 23 days after the surgery with no antifungal treatment. Approximately 4 years after the operation, she is in good health without any signs of fungal infection.

**Fig. 5:**
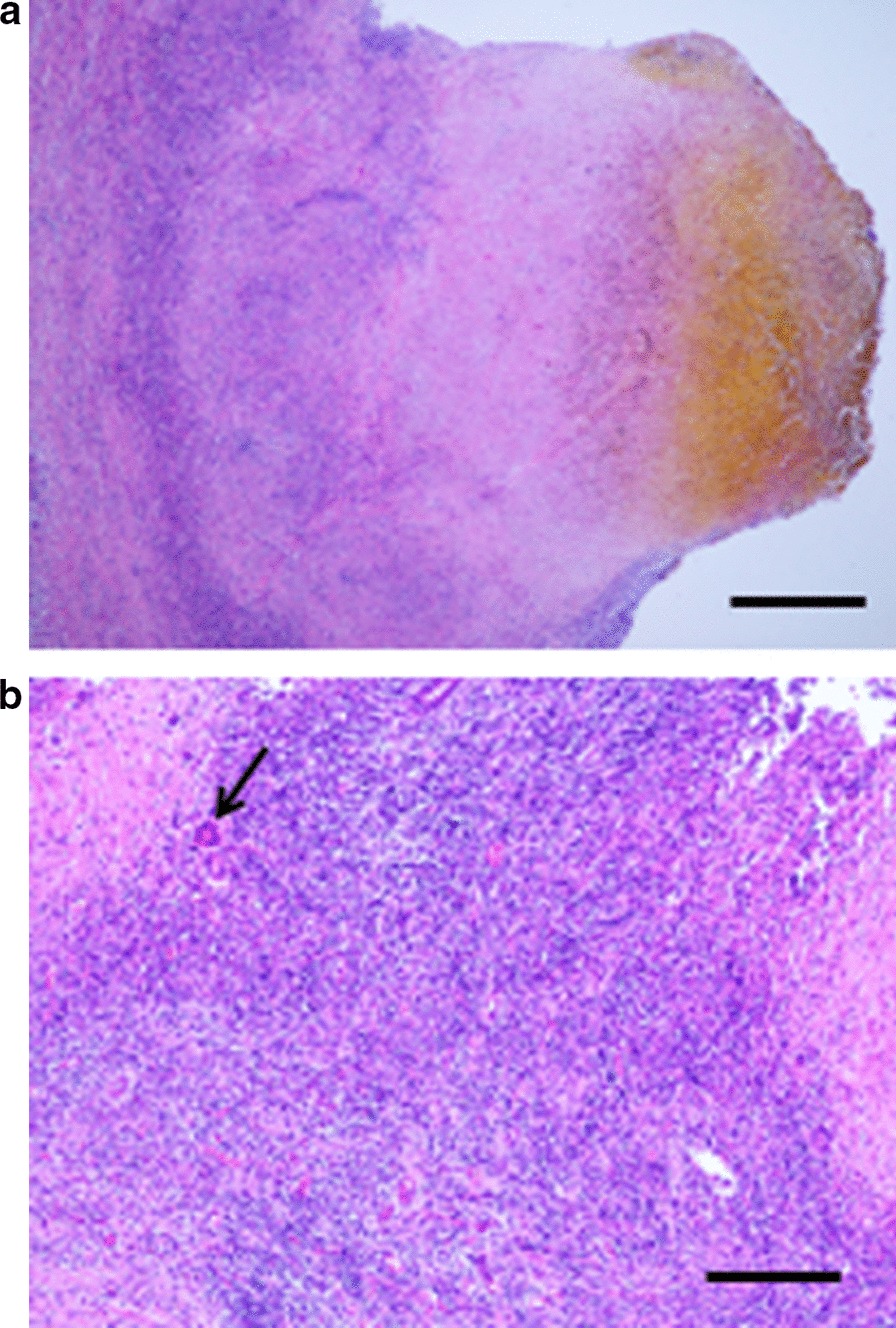
Histopathological findings. **a** Granulomatous cholangitis with necrosis accompanied by bile duct thickening (scale bar = 500 μm). **b** Multinucleated giant cells (arrow) (scale bar = 200 μm)

**Fig. 6:**
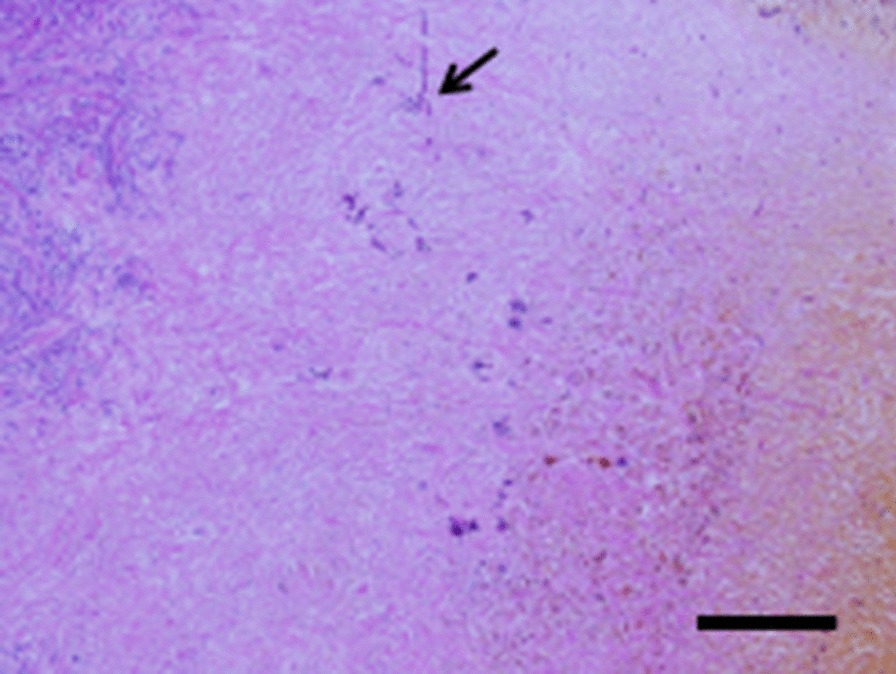
Histopathological findings of periodic acid–Schiff (PAS) staining. Fungal infection with granulomatous cholangitis. PAS can be used to visualize some fungal organisms in tissue sections (arrow) (scale bar = 200 μm)

Histologically, a great number of large granulomatous lesions were found in the left and right hepatic ducts, their confluence, and the lower bile duct, and necrotic tissue was formed in the central part (Fig. 5a). Multinucleated giant cells (Fig. 5b, arrow) were also observed. Infiltration of inflammatory cells and fibrosis were observed around the granuloma, and an associated thickening of the bile duct wall was found remarkable. Erosion and ulceration were noticeable in the bile duct lumen. No obvious malignant findings were noted. Histopathological findings consisted of granulomatous cholangitis with necrosis, accompanied by bile duct thickening. In the necrotic tissue of the central part of the granuloma, Periodic acid–Schiff (PAS) stains positive structures that consider yeast and pseudohyphae are scattered (Fig. 6). This finding was consistent with fungus, with unclear branches and septa, and was considered to be Candida. Because Ziehl–Neelsen staining was negative, we ruled out infection with acid-fast bacillus. The final histopathological diagnosis of the surgical specimens showed granulomatous cholangitis based on fungal infection.

## Discussion and conclusions

Because the curative operation for hilar bile duct tumor can result in an excessively invasive extended resection, utmost care must be taken in order to determine if the lesion is benign or malignant. However, even with the recent advances in diagnostic imaging, hilar bile duct stenosis, in which it is difficult to distinguish between benign and malignant, is not uncommon. In addition, preoperative histopathological assessment with biopsy or biliary cytology has been reported to have limited sensitivity [2, 3]. Benign disease has been found to account for 14.1% of resected cases [4]. In our case, preoperative imaging indicated a suspicion for stenosis, and, because of the tumor in the hilar bile duct, we performed the surgery. However, the permanent histopathological findings revealed that the resected specimen was benign. Furthermore, we opted to avoid extended invasive resection by limiting the extent of the resection to the extrahepatic bile duct and confirming the negative stump using rapid intraoperative diagnosis.

Granulomatous cholangitis was the final diagnosis of our case, based on fungal infection. Diseases that form a granuloma in the hilum of the liver include primary biliary cirrhosis, drug-induced liver injury, tuberculosis, sarcoidosis, cytomegalovirus, Epstein-Barr virus infection, and so forth [5].

Few cases have been reported in which the resected tumor of the hilum, which could not be ruled out as malignant, was attributed to a fungal infection [6]. Thus, our case is considered as a rare resected case, in which tumor in the hilum of the liver was found to be due to fungal infection. Although infections with Candida and other fungal species have been increasingly recognized in patients with certain predispositions leading to impaired immune function, fungal involvement of the biliary tract has rarely been observed, even in susceptible patients with systemic candidiasis. Recently, there has been an increase in biliary fungal involvement, and obstructive jaundice resulting from biliary fungal infections has increasingly been recognized in the past few years [1]. As many of these patients suffer not only from biliary mycosis but also from diseases requiring immunosuppression, the prognosis can be deemed poor. In general, anti-infectious drug treatment as the main therapy for biliary candidiasis should be complemented by endoscopic intervention [1]. Although our patient had a history of chondrodystrophy, she was considered to be an immunocompetent adult. Because she was not treated with immunosuppressants or steroids, we never believed that she had a systemic fungal infection. Furthermore, we never assumed that she had an isolated fungal infection, which is deemed to be rarer than a systemic fungal infection. Fungal infection should be considered one of the differential diagnoses, even if the patient is immunocompetent.

In conclusion, isolated fungal infections in the hilar bile ducts in immunocompetent patients are considered to be rare and difficult to diagnose from the outset. Hilar cholangiocarcinoma is likely to require expanded surgery to ensure its curability. The diagnosis of benign or malignant is difficult; in our case, we did not immediately consider extended liver resection. Because our case had a developmental disorder, we tried to avoid excessive invasion, but, even in patients without developmental disorders, it is desirable to avoid excessive invasion if there is a possibility of benign biliary stenosis. In our strategies, if benign biliary stenosis cannot be ruled out, we consider that a local resection is performed first. And then, rapid intraoperative diagnosis is performed, and if the result is positive for the bile duct stump on the liver, extended hepatectomy is performed. As in our case, the dissection of the hepatoduodenal ligament, resection of the extrahepatic bile duct, and rapid intraoperative pathological examination should be performed before deciding to perform extended liver resection.

## Data Availability

All available data are presented in the case.

## Abbreviations

ENBD: Endoscopic nasobiliary drainage
PAS: Periodic acid–Schiff
